# Response to Letter to the Editor: Importance of considering high-risk behaviours in COVID-19 vaccine effectiveness estimates with observational studies

**DOI:** 10.2807/1560-7917.ES.2023.28.4.2300044

**Published:** 2023-01-26

**Authors:** Catharina E van Ewijk, Susan JM Hahné, Mirjam J Knol, on behalf of all co-authors of the original article

**Affiliations:** 1 Centre for Infectious Disease Control, National Institute for Public Health and Environment (RIVM), Bilthoven, the Netherlands; 2 European Programme for Intervention Epidemiology Training (EPIET), European Centre for Disease Prevention and Control (ECDC), Stockholm, Sweden; 3 https://www.eurosurveillance.org/content/10.2807/1560-7917.ES.2022.27.45.2200217

**Keywords:** COVID-19 vaccine effectiveness, risk-behaviour, confounding, test-negative design

**To the editor:** We thank Arashiro et al. for their interesting comments on our study in which we concluded that collecting data on risk behaviour in a test-negative COVID-19 vaccine effectiveness study is not necessary in the Dutch population. A concern by Arashiro et al. was whether this conclusion was fully supported by our results.

Firstly, we fully agree that it remains crucial to carefully consider potential bias and confounders when estimating vaccine effectiveness (VE). As pointed out, bias and confounders are context-specific, and we must be cautious to generalise findings from one setting to the other.

We therefore want to stress that our main message is not to discourage addressing confounding in VE studies, but to demonstrate that confounding by behaviour, at least in a test-negative case–control study, might have less impact in practice than expected based on theory [1]. This information can be valuable since collecting data on behaviour might be time-consuming and expensive, and therefore often not feasible.

Differential measures, such as access to events, and therefore differential risk of exposure for vaccinated compared with unvaccinated individuals are likely to confound VE estimates. As mentioned, the Netherlands implemented a ‘coronavirus entry pass’ as of 25 September 2021. Contrary to what Arashiro et al. suspect, this coronavirus entry pass could also be obtained by unvaccinated individuals. An entry pass was given by showing proof of either complete vaccination, recovery after COVID-19, or a negative test result taken less than 24 h before entry. Testing was free of charge and readily available throughout the Netherlands at various locations. The coronavirus entry pass was mandatory to access almost all venues including restaurants, cafés, festivals, theatres, cinemas and other events such as sport events [2]. In addition, we captured behaviour that did not require a coronavirus entry pass, such as e.g. participating in outdoors demonstrations. Since the coronavirus entry pass was implemented for the general public irrespective of vaccination status, we believe that this measure did not majorly confound our VE estimates or would explain the waning immunity that was observed.

A concern remains, however, whether our results can be generalised to the whole population. As we pointed out in our discussion, there might be selection bias present. It is possible that with our sampled population (people coming forward for testing), we did not capture individuals who were at highest risk of SARS-CoV-2 exposure through their behaviour, and that we oversampled individuals who are more likely to adhere to non-pharmaceutical interventions, which would probably lead to an underestimation of the effect of behaviour as confounder.

Arashiro et al. did not find a major difference in COVID-19 VE estimates when adjusting for behaviour and thus chance of SARS-CoV-2 exposure in their setting with no differential measures for vaccinated and unvaccinated individuals. We also did not find large effects of adjusting for behaviour in our setting with a coronavirus entry pass which could be obtained by vaccinated and unvaccinated individuals. This shows that behaviour might not always have a major confounding impact depending on setting and the non-pharmaceutical interventions in place [3].

Nonetheless, critical evaluation of potential biases and confounders, that might differ per setting, remain essential to prevent unwarranted interpretations and generalisation of VE estimates.
